# Antibody response against SARS-CoV-2 variants of concern in children infected with pre-Omicron variants: An observational cohort study

**DOI:** 10.1016/j.ebiom.2022.104230

**Published:** 2022-08-18

**Authors:** Vanesa Seery, Silvina Raiden, Constanza Russo, Mauricio Borda, Largión Herrera, Macarena Uranga, Augusto Varese, María Marcó del Pont, Carina Chirino, Constanza Erramuspe, Laura Silvana Álvarez, Melisa Lenoir, Laura Daniela Morales, Carolina Davenport, Alexsa Alarcón Flores, Soledad Huespe Auchter, Yanina Ruiz, Liliana Monsalvo, Laura Sastoque, Magalí Gavazzi, Ignacio Mazzitelli, Facundo Di Diego, Yesica Longueira, Bianca Mazzitelli, Inés Sananez, Norberto De Carli, Mirna Marcela Biglione, Juan Martín Gómez Penedo, Ana Ceballos, Natalia Laufer, Fernando Ferrero, Jorge Geffner, Lourdes Arruvito

**Affiliations:** a Instituto de Investigaciones Biomédicas en Retrovirus y SIDA (INBIRS). Facultad de Medicina. UBA-CONICET, Paraguay 2155, C1121ABG Caba, Argentina; b Hospital General de Niños Pedro de Elizalde, Av. Montes de Oca 40, C1270 CABA, Argentina; c Hospital Pediátrico Juan Pablo II, Av. Artigas 1435, W3400 Corrientes, Argentina; d Hospital Dr. Salvador Mazza, Sta. Josefa Rosello 356, H3540 Chaco, Argentina; e Hospital Universitario Austral, Av. Juan Domingo Perón 1500, B1629 Buenos Aires, Argentina; f Policlínico Regional Juan Domingo Perón, Maipú 450, D5732 San Luis, Argentina; g Clínica del Niño de Quilmes, Av. Lamadrid 444, B1878 Buenos Aires, Argentina; h Facultad de Psicología, UBA- CONICET, Av. Hipólito Yrigoyen 3242, C1207ABR Caba, Argentina

**Keywords:** Pediatric COVID-19, SARS-CoV-2, Variants, Vaccines, Antibodies

## Abstract

**Background:**

Despite that pediatric COVID-19 is usually asymptomatic or mild, SARS-CoV-2 infection typically results in the development of an antibody response. Contradictory observations have been reported when the antibody response of children and adults were compared in terms of strength, specificity and perdurability.

**Methods:**

This observational study includes three cohorts infected with SARS-CoV-2 between March 2020-July 2021: unvaccinated infected children (*n=*115), unvaccinated infected adults (*n=*62), and vaccinated infected children (*n=*76). Plasma anti-spike IgG antibodies and neutralising activity against Wuhan, Delta and Omicron variants after 7-17 months post-infection were analysed.

**Findings:**

More than 95% of unvaccinated infected children and adults remained seropositive when evaluated at 382-491 and 386-420 days after infection, respectively. Anti-spike IgG titers and plasma neutralising activity against Wuhan, Delta and Omicron variants were higher in children compared to adults. No differences were found when unvaccinated infected children were stratified by age, gender or presence/absence of symptoms in the acute phase of SARS-CoV-2 infection, but a slight decrease in the antibody response was observed in those with comorbidities. Vaccination of previously infected children with two doses of the inactivated BBIBP-CorV or the mRNA vaccines, BNT162b2 and/or mRNA-1273, further increased anti-spike IgG titers and neutralising activity against Wuhan, Delta and Omicron variants.

**Interpretation:**

Unvaccinated infected children mount a more potent and sustained antibody response compared with adults, which is significantly increased after vaccination. Further studies including not only the analysis of the immune response but also the effectiveness to prevent reinfections by the different Omicron lineages are required to optimise vaccination strategy in children.

**Funding:**

National Agency for Scientific and Technological Promotion from Argentina (PICTO-COVID-SECUELAS-00007 and PMO-BID-PICT2018-2548).

**Research in context**

**Evidence before this study**

Children and youth infected with SARS-CoV-2 typically show an asymptomatic or mild disease. This contrasts with other viral respiratory infections which can induce a severe disease, especially in young infants. It has been suggested that children develop an earlier and more robust innate immune response upon SARS-CoV-2 infection compared with adults. The possible contribution of a more efficient adaptive immune response to the favourable outcome of pediatric COVID-19 has not yet been determined.

**Added value of this study**

Here, we show that in response to SARS-CoV-2 infection, children developed a higher and more sustained antibody response compared with adults. When measured at 7-17 months after infection, both the titres of IgG antibodies directed to the spike protein of SARS-CoV-2, and the plasma neutralising activity against the Wuhan original variant and the variants of concern Delta and Omicron were higher in children compared with adults. We also found that vaccination with two-doses of the inactivated BBIBP-CorV vaccine or the mRNA vaccines BNT162b2 and mRNA-1273 further increased the plasma neutralising activity against the variants of concern Delta and Omicron in previously infected children.

**Implications of all the available evidence**

The special signature of the antibody response in children upon SARS-CoV-2 infection may provide some degree of immune advantage compared to adults in terms of adaptive immunity. This observation together with the ability of the current anti-SARS-CoV-2 vaccines to further enhance the antibody response against VOCs including Delta and Omicron in previously infected children, may inform vaccination strategies in the pediatric population.

Alt-text: Unlabelled box

## Introduction

Children and adolescents usually exhibit an asymptomatic or mild disease after severe acute respiratory syndrome coronavirus 2 (SARS-CoV-2) infection.^1^, ^2^, ^3^ However, they can also suffer severe Coronavirus disease 2019 (COVID-19). Indeed, children with comorbidities have shown a higher risk of severe COVID-19 compared with those without underlying disease.^4^ Moreover, in rare cases, infected children can develop an acute multisystem inflammatory syndrome (MIS-C) several weeks after the resolution of acute infection.^5^ Even though the number of pediatric patients with COVID-19 increased after the spread of variants with greater transmissibility, like Delta and Omicron,^6^ children show a lower severity and mortality compared to adults.^7^ Recent studies have suggested that children display an earlier and robust innate immune response upon SARS-CoV-2 infection.^8^, ^9^, ^10^ The possible contribution of a more efficient adaptive immune response to the favourable outcome of pediatric COVID-19 has not been clarified yet.

Different studies have analysed the antibody response elicited in children upon SARS-CoV-2 infection. Weisberg and coworkers^11^ reported that the antibody response in adults is characterised by the production of anti-spike and anti-nucleocapsid IgG antibodies while children only produce IgG antibodies directed to the spike protein showing a lower neutralising activity. We have previously reported that children with severe COVID-19 display a weaker and delayed kinetic of antibody response compared with those with asymptomatic or mild disease.^12^ Three recent studies have analysed the persistence of the antibodies in children after SARS-CoV-2 infection. Garrido and coworkers^13^ reported a robust IgM, IgG and IgA antibody response to a broad array of SARS-CoV-2 antigens in children with asymptomatic or mild disease for at least 4 months post infection. Dowell and coworkers^14^ reported that children develop a robust and sustained antibody response with higher levels of anti-spike IgG antibodies but similar serum neutralising activity against variant of concerns (VOCs), compared with adults. Consistent with these observations, Renk and coworkers^15^ showed that children display a stronger antibody response compared with adults that persist at 11-12 months after infection.

COVID-19 vaccines are now being administered widely to children in several countries.^16^, ^17^, ^18^ In Argentina, vaccination with mRNA vaccines BNT162b2 (Pfizer/BioNTech) and mRNA-1273 (Moderna) has been approved for children and adolescents aged between 12 and 17 years in August 2021. Moreover, vaccination with a whole-cell inactivated vaccine (BBIBP-CorV, Sinopharm) was approved for its use in children aged between 3 and 11 years last October 2021 (https://bancos.salud.gob.ar/recurso/informe-especial-de-vigilancia-de-seguridad-en-vacunas-en-ninos-ninas-y-adolescentes).

In this work, we analysed the anti-spike IgG titres and the plasma neutralising activity against the ancestral variant (Wuhan) and the VOCs Delta and Omicron in a cohort of 115 unvaccinated infected children and 62 unvaccinated infected adults up to 17 months after SARS-CoV-2 infection. In addition, we analysed the antibody response elicited by vaccination with BBIBP-CorV, BNT162b2 and mRNA-1273 vaccines in a cohort of 76 previously infected children during the pre-Omicron phase of the pandemic.

## Methods

### Study population

Two pediatric cohorts were enrolled in this observational study, that followed The Strengthening the Reporting of Observational studies in Epidemiology (STROBE) guidelines. Both pediatric cohorts were enrolled months after having acute COVID-19 (up to 17 months post-infection). Children were initially admitted to the Hospital General de Niños Pedro de Elizalde, Hospital Universitario Austral, Hospital Dr. Salvador Mazza, Hospital Pediátrico Juan Pablo II, Policlínico Regional Juan Domingo Perón and Clínica del Niño de Quilmes, in the course of acute infection, between March 2020 and July 2021, when the variants Wuhan (B1), Alpha (UK), Gamma (Manaos), Lamba (Peru) or Mu (Colombia) circulated in Argentina (http://pais.qb.fcen.uba.ar/reports.php).^19^ The first cohort consisted of 115 girls, boys and adolescents with previous SARS-CoV-2 infection diagnosed between March 2020 and July 2021 and unvaccinated status. The median age and IQR of unvaccinated infected children group was 2 years (1-8), of whom 44% (*n=*50) were girls. The median time since COVID-19 diagnosis until sampling was 447 days (IQR, 382-491). Sixty percent of children (*n=*69) had no comorbidities, and respiratory disease was the most prevalent underlying problem (21%, *n=*24) in those with comorbidities. According to the criteria from WHO, all children had asymptomatic or mild disease in the course of SARS-CoV-2 infection. It is important to mention that in Argentina, all pediatric patients with confirmed diagnosis of SARS-CoV-2 infection (even if they had a mild condition or were asymptomatic) were hospitalised during the first wave between July and December, 2020. Three out of 115 unvaccinated infected children required ICU admission during the acute COVID-19 due to underlying diseases and not as a consequence of SARS-CoV-2 infection: 1 had Down syndrome, 1 had bronchopulmonary dysplasia and epilepsy and 1 received ICU treatment after shunt surgery for hydrocephalus. The second cohort consisted of 76 vaccinated previously infected children. Children and adolescents included in this cohort received one- or two-doses of BBIBP-CorV (*n=*36) or the mRNA vaccines BNT162b2 and mRNA-1273 (*n=*40), after SARS-CoV-2 infection between March 2020 and July 2021. The median age and IQR of vaccinated infected cohort was 12 years (9-15) and 63% (*n=*48) were girls. The median time since COVID-19 diagnosis until sampling was 316 days (IQR, 205-496). The median time since the first dose until blood collection was 23 days (IQR, 17-30) and regarding the second dose was 45 days (IQR, 31-68). Sixty-one percent (*n=*46) of vaccinated infected children were previously healthy, with respiratory disease (*n=*11) and obesity (*n=*6) being the most frequent comorbidities. Children were asymptomatic or mildly affected in the course of SARS-CoV-2 infection. Only 1 vaccinated infected child required ICU admission at the time of diagnosis due to diabetic ketoacidosis. All of the children included in both cohorts had been discharged and were fully recovered at enrolment (up to 17 months post infection). Children that after their primary infection with SARS-CoV-2 had a new confirmed SARS-CoV-2 re-infection and/or those in close-contact with infected individuals were excluded. At the time of sampling, none of children was hospitalised, suffered any active acute infection or received antiviral or steroid treatment. A third cohort, recruited at the Biobanco de Enfermedades Infecciosas (INBIRS, Facultad de Medicina-UBA-CONICET), included 62 unvaccinated infected adults, the median age and IQR was 35 years (30-47), and 55% (*n=*34) were women. The median time since COVID-19 diagnosis (between March 2020 and June 2020) until sampling was 391 days (IQR, 386-420). Most of them (89%) had no comorbidities. All adults had mild disease. None of them was hospitalised in the course of acute infection or suffered any active infection or received antiviral or steroid treatment at the time of sampling. Table 1 summarises the main characteristics of all the children and adults included in the study. Table S1 shows a detailed description of the vaccinated infected children cohort. Figure S1 shows the frequency of circulating SARS-CoV-2 variants in Argentina over the period 2020-2021, and the time at which the included individuals were infected by SARS-CoV-2.

**Table 1:** Characteristics of study cohorts.

	Children		Adults
	Unvaccinated infected (*n=*115)	Vaccinated infected (*n=*76)	Unvaccinated infected (*n=*62)
Age, years, median (range)	2 (1-8)	12 (9-15)	35 (30-47)
<2, n (%)	44 (38)	0	N/A
2-5, n (%)	30 (26)	12 (16)	N/A
6-11, n (%)	26 (23)	24 (32)	N/A
12-17, n (%)	15 (13)	40 (52)	N/A
≥18, n (%)	N/A	N/A	62 (100)
Female, n (%)	50 (44)	48 (63)	34 (55)
COVID-19 Diagnostic			
PCR positive, n (%)	111 (96)	61 (80)	62 (100)
Rapid Antigen Test positive, n (%)	4 (4)	15 (20)	0
Severity of illness^a^, n (%)			
Asymptomatic	26 (23)	12 (16)	0
Mild	89 (77)	64 (84)	62 (100)
SARS-CoV-2 IgG antibody positive^b^, n (%)	110 (96)	76 (100)	60 (97)
Days post infection^b^, median (range)	447 (382-491)	316 (205-496)	391 (386-420)
Symptomatic^a^, n (%)	89 (77)	64 (84)	62 (100)
Comorbidities^c^, n (%)			
None	69 (60)	46 (61)	55 (89)
Prematurity	6 (5)	4 (5)	0
Respiratory disease	24 (21)	11 (14)	1 (2)
Heart disease	6 (5)	3 (4)	2 (3)
Renal disease	5 (4)	2 (3)	0
Neurological disease	11 (10)	4 (5)	0
Obesity	5 (4)	6 (8)	2 (3)
Undernutrition	3 (3)	4 (5)	0
Cancer	4 (4)	2 (3)	1 (2)
Diabetes	1 (1)	1 (1)	0
Genetic disorder	3 (3)	1 (1)	0
Autoimmunity	2 (2)	0	3 (5)
ICU admission^a^, n (%)	3 (3)	1 (1)	0
Vaccination			
BBIBP-CorV, n (%)			
1 dose	N/A	17 (22)	N/A
2 doses	N/A	19 (25)	N/A
BNT162b2 / mRNA-1273, n (%)			
1 dose	N/A	13 (17)	N/A
2 doses	N/A	27 (36)	N/A
Days post first dose^b^, median (range)	N/A	23 (17-30)	N/A
Days post second dose^b^, median (range)	N/A	45 (31-68)	N/A

a At acute COVID-19;

b At time of sampling;

c Comorbidities include prematurity, respiratory disease (asthma, recurrent weezing, bronchiolitis, bronchopulmonary dysplasia, croup, cystic fibrosis), heart disease (congenital cardiopathy), renal disease (chronic kidney disease), neurological disease (epilepsy, hydrocephalus), obesity, undernutrition, cancer (retinoblastoma, liver cancer), diabetes, genetic disorder (Down syndrome), autoimmunity (idiopathic thrombocytopenic purpura, autoimmune hemolytic anemia).

### Study approval

This study was conducted in accordance with the Declaration of Helsinki. The Institutional Review Board from institutions participants reviewed and approved the sample collection and the overall study (Hospital General de Niños Pedro de Elizalde #1226/20, Hospital Universitario Austral #P21-064 and IATIMET Universidad de Buenos Aires #1.0/150621). Sample collection from the Biobanco de Enfermedades Infecciosas was approved by the Fundación Huésped Bioethics Committee.

### Ethics

Adults provided written informed consent for the donation of samples to the Biobanco de Enfermedades Infecciosas. Parents or legal guardians from children under 8 years provided written, informed consent. Children older than 8 years old provided written, informed consent and their parents or legal guardians also provided written, informed consent. All samples were deidentified prior to processing.

### Blood sample processing

Approximately 0.5-1 mL of whole blood samples were obtained. After being centrifuged for 10 min at 1000 rpm, plasma was separated and stored at −80°C until used.

### Cells and virus

VERO C1008 (clone E6, ATCC, RRID:CVCL_0574) were cultured in DMEM (GIBCO, 10313021) supplemented with 5% heat-inactivated fetal bovine serum (FCS, Sigma-Aldrich, F2442), 2mM L-Glutamine (Sigma-Aldrich, G7513), penicillin and streptomycin (Sigma-Aldrich, P0781). Cells were incubated in 95% air and 5% CO_2_ at 37°C. The Wuhan variant isolate (hCoV-19/Argentina/PAIS-G0001/2020, GISAID Accession ID: EPI_ISL_499083) was kindly provided by Dr. Sandra Gallegos (InViV working group, Universidad Nacional de Córdoba, Argentina). The Delta (GISAID Accession ID: EPI_ISL_11014871) and Omicron (GISAID Accession ID: EPI_ISL_10633761) variants were isolated at INBIRS (Facultad de Medicina, UBA-CONICET, Argentina) from samples of nasopharyngeal swabs. Viral master seed stock was prepared using T75 flasks of Vero E6 cells. Each flask was harvested on day two post infection, and the supernatant was centrifuged twice at 220 x g for 15 min to remove cellular debris. The virus stock titre was determined by plaque assay on Vero E6 cells and expressed as plaque-forming units per mL. Viral stock identity was confirmed by whole-genome sequencing in an Illumina sequencer. The experiments using the virus were carried out in BSL3 facilities at INBIRS (Facultad de Medicina, UBA-CONICET, Argentina).

### Quantitation of plasma SARS-CoV-2–specific IgG antibodies

IgG antibodies to SARS-CoV-2 spike protein were detected using an established commercially available two-step ELISA (COVIDAR), as described.^20^ Briefly, the assay uses 96-wells plates coated with a mixture of spike and the receptor binding domain (RBD). The viral proteins were purified from transfected FreeStyle 293-F (RRID:CVCL_D603) cells using HisTrap excel columns. The horseradish peroxidase (HRP)-conjugated monoclonal antibody used for human IgG detection in the COVIDAR ELISA was G18-145, which specifically binds to the heavy chain of all four human immunoglobulin G subclasses: IgG1, IgG2, IgG3, and IgG4. Deindentified plasma samples were diluted in PBS-T containing 0.8% casein and incubated for 1 h at 37°C on spike and RBD-coated plates. For endpoint titration, plasma samples were two-fold serially diluted in FCS, added to the plates and incubated for 1 h at 37°C. A 10-point curve with each point done in duplicate was assessed (1/10 to 1/5120) per sample. After washing with PBS-T, HRP-conjugated goat mouse anti-human IgG antibodies were added and incubated for 30 min at 37°C, followed by TMB Substrate Reagent. The absorbance (OD) was measured at 450 nm in a SpectraMax i3 plate reader (Molecular Devices). The positive (OD >1) and negative (OD <0.15) sera controls provided were run in duplicate. The cut-off was calculated as the mean OD of the negative sera plus 0.15. Samples with OD lower than the cut-off were considered negative. The anti-spike IgG antibody titres were determined by endpoint titration defined as the reciprocal of the highest dilution of a serum that gives a reading above the cut-off.

### Neutralisation assay

Neutralisation assays were performed as described by Rossi et al.^21^ Briefly, deindentified plasma samples were heat-inactivated at 56°C for 20 min and 2-fold serial dilutions were mixed at 37°C for 1 h, with the Wuhan (MOI=0.01), Delta (MOI=0.01) and Omicron variants (MOI=0.01) in DMEM 2% FBS. Fifty µL of these mixtures were then deposited over Vero E6 cell monolayers for 1 h at 37°C in 96-well plate. Infectious media was then removed and replaced with DMEM supplemented with 2% FCS. After 72 h of culture, cells were fixed with 4% paraformaldehyde (Sigma–Aldrich, 158127) at 4°C during 20 min and stained with crystal violet solution in methanol (Sigma–Aldrich, 34860) at room temperature for 5 min. A 12-point serial dilution curve with each point done in duplicate was assessed (1/8 to 1/16384) per sample. Controls without plasma or without virus were performed and an internal standard of known neutralisation capacity were run in parallel to ensure reproducibility. Eighty reads per well absorbance at 585 nm was measured in a SpectraMax i3 plate reader (Molecular Devices). Inhibitory concentrations of 50% (IC50) values were calculated for all patient plasma samples by modelling a 4-parameter logistic regression with GraphPad Prism 8. This model describes the sigmoid-shaped response pattern. The IC50 was calculated as the point at which the curve matches an inhibition of 50%.

### Statistics

Clinical characteristics were summarised using descriptive statistics. Categorical variables are reported as numbers and percentages. Quantitative variables are reported as medians and interquartile ranges and presented as medians and minimum to maximum in the figures. The normality of experimental data was evaluated by the Shapiro–Wilk test. Two groups were compared using the Wilcoxon signed-rank test or Mann-Whitney U test. Two groups proportions were compared using the Chi-square test. Three or more groups were compared using the Kruskall–Wallis test followed by Dunn's multiple comparison test (the method used is stated in the figure legends). Correlation between two continuous variables was calculated using a Spearman correlation test. To conduct the regression model for the titres of IgG estimating group effects, while adjusting for gender, days post infection and presence/absence of comorbidities was used the software environment R (R Core Team, 2021). Statistical significances are indicated in the figures by asterisks as follows: *p< 0.05, **p< 0.01, ***p< 0.001 or ****p< 0.0001. Analysis and visualizations were performed using GraphPad Prism.v.8 (GraphPad Software) and the software environment R.

### Role of funders

The funders had no role in study design, data collection, analysis and interpretation, writing and submission of the manuscript.

## Results

### Unvaccinated children develop a robust and durable antibody response up to 17 months after SARS-CoV-2 infection

**Figure 1:**
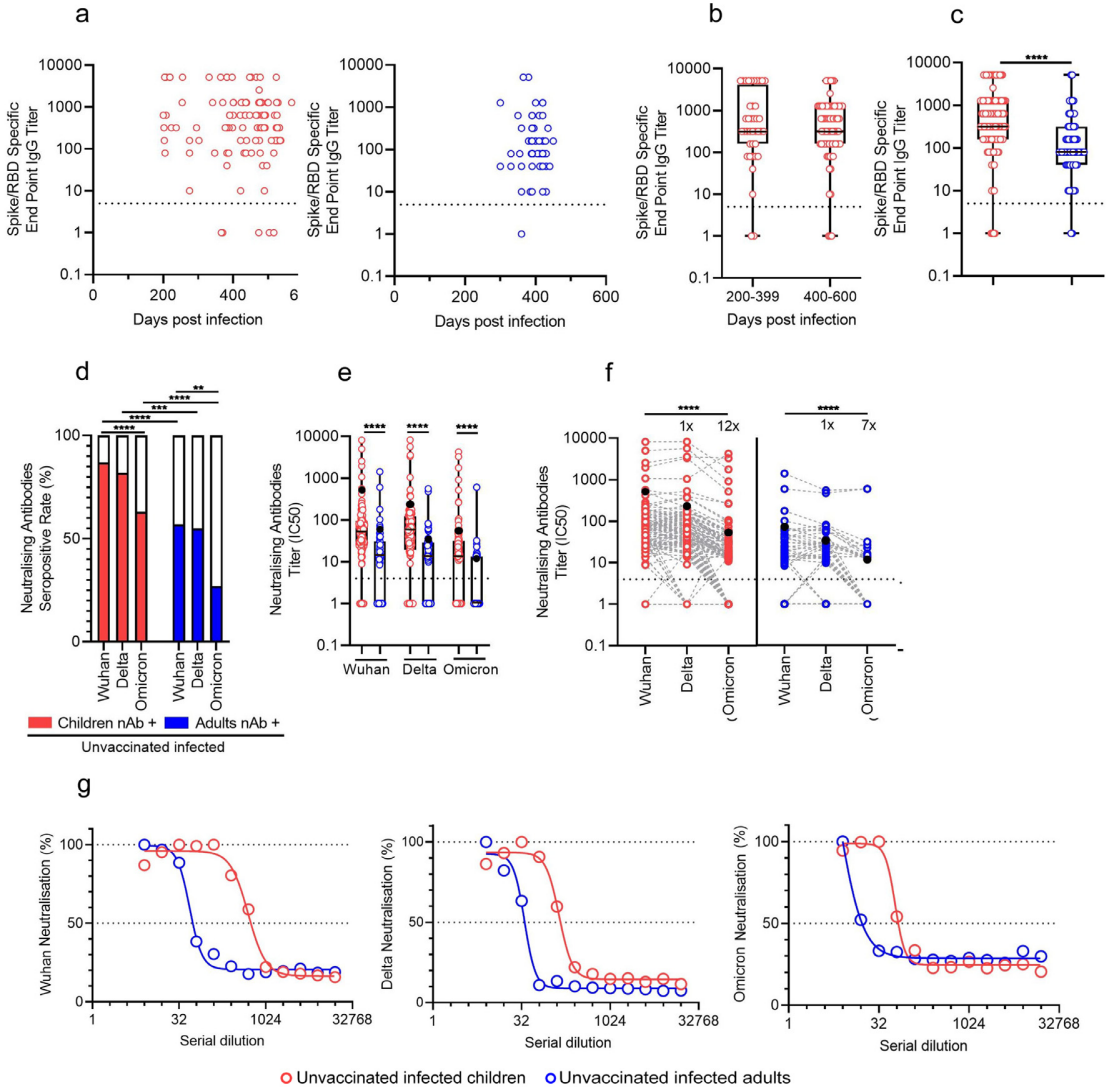
**Persistence of the antibody response in unvaccinated infected children and adults. (a-c)** Titres of IgG anti-spike antibodies defined by end point dilution in plasma from unvaccinated infected children (*n=*115) and unvaccinated infected adults (*n=*62). **(a)** Titres of IgG anti-spike antibodies plotted over days post infection (children, left panel; adults right panel). **(b)** Titres of IgG anti-spike antibodies in unvaccinated infected children grouped according days post infection (200 to 399 days, *n=*40) and 400 to 600 days (*n=*75). **(c)** Comparison of IgG anti-spike titres between unvaccinated infected children and unvaccinated infected adults. **(d-e)** Neutralisation activity against Wuhan, Delta and Omicron variants in plasma from unvaccinated infected children and unvaccinated infected adults. **(d)** Bar graphs showing the percentage of positive samples for neutralising activity against Wuhan, Delta and Omicron variants. **(e)** Neutralisation antibody titres against Wuhan, Delta and Omicron variants determined by the reciprocal IC50 in plasma from unvaccinated infected children and unvaccinated infected adults. **(f)** Comparison of neutralisation antibody titres against the three variants in paired samples. Fold decrease was calculated dividing the Wuhan IC50 by the Delta or Omicron IC50. **(g)** Representative curves of neutralising activity against Wuhan, Delta and Omicron variants in plasma from an unvaccinated infected child and an unvaccinated infected adult. These donors are shown in e and f as black filled dots. Dotted line indicates the limit of detection value. Median and min to max of n donors are shown in b, c and e. P values were determined by Pearson's Chi square test and Mann–Whitney U test: ** *p<*0.01, *** *p<*0.001, **** *p<*0.0001. Unvaccinated infected children (red circle), unvaccinated infected adults (blue circle).

Initially, we analysed the plasma levels of IgG antibodies directed to the spike protein of SARS-CoV-2 in samples obtained from 115 unvaccinated infected children and 62 unvaccinated infected adults. The median time (IQR) of sampling was 447 days (382-491) and 391 days (386-420) after diagnosis, for children and adults respectively. We found that more than 95% of both children and adults remained seropositive (Figure 1a and Table 1). The titres of IgG anti-SARS-CoV-2 antibodies in unvaccinated infected children were similar when evaluated after 200-399 days (*n=*40) and 400-600 days (*n=*75), after infection (Figure 1b). Interestingly, titres of IgG antibodies to the spike protein of SARS-CoV-2 were higher for unvaccinated infected children compared with unvaccinated infected adults [320 (160-1280), 80 (40-320), *p<*0.0001; median (IQR) in children and adults, respectively, Figure 1c]. When unvaccinated infected children were stratified according to their age, gender and presence or absence of symptoms at COVID-19 diagnosis no differences were found (Figure S2. a-c). Plasma levels of anti-spike IgG antibodies were lower in unvaccinated infected children with comorbidities (Figure S2.d). In order to compare the titres of anti-SARS-CoV-2 IgG antibodies between unvaccinated infected children and adults adjusting for variables such as gender, days post infection and presence/absence of comorbidities, we performed a linear regression test including these variables as covariates. This regression model adjusting for the covariates confirmed a significant difference in the titres of anti-spike IgG antibodies between children and adults [860.10 (385.46-1334.73, 95% CI), *p<*0.001].

### Unvaccinated children infected by SARS-CoV-2 before the emergence of the Omicron variant develop a neutralising antibody response against Omicron

We then studied the plasma neutralising activity against the SARS-CoV-2 ancestral Wuhan variant, and the VOCs Delta and Omicron. As described above, plasma samples were obtained in all cases within a median time of 447 or 391 days after infection diagnosis from unvaccinated infected children and adults respectively, who got infected between March 2020 and July 2021, before the emergence of the Omicron variant.^22^ In our country, Omicron variant was detected for the first time in December 2021. Eighty-seven percent (*n=*100) and 82% (*n=*94) of unvaccinated infected children were shown to be positive for the presence of plasma neutralising antibodies against Wuhan and Delta variants, respectively. Of note, 63% (*n=*72) of these children were positive for the presence of neutralising antibodies against the Omicron variant. For all the variants analysed, unvaccinated infected adults showed lower percentages of positive samples, decreasing to 27% (*n=*17) for the Omicron variant (Figure 1d). Not only the rate of seropositivity was higher in unvaccinated infected children compared with adults, but also the titres of neutralising antibodies [52 (24-94), 55 (19-122) and 14 (1-32) vs 12 (1-31), 12 (1-29) and 1 (1-13), median (IQR), for Wuhan, Delta and Omicron variants in children and adults respectively (*p<*0.0001); Figure 1e]. As expected, analysis of paired samples confirmed a strong reduction in the neutralising titres against Omicron compared with the Wuhan variant in both, unvaccinated infected children [52 (24-94) and 14 (1-32), median (IQR), for Wuhan and Omicron variants, respectively, *p<*0.0001] and adults [12 (1-31) and 1 (1-13), median (IQR), for Wuhan and Omicron variants, respectively, *p<*0.0001, Figure 1f]. Representative neutralisation curves against the three variants by plasma samples from an unvaccinated infected child and an unvaccinated infected adult are shown in Figure 1g. In addition, a clear correlation [Spearman r (95% confidence interval] was found when the titres of anti-spike IgG antibodies and neutralising antibodies against the three variants were analysed in samples from either unvaccinated children [0.84 (0.77-0.89), 0.75 (0.66-0.83) and 0.66 (0.55-0.76) for Wuhan Delta and Omicron variants, respectively, *p<*0.0001] or unvaccinated infected adults [0.67 (0.51-0.80), 0.60 (0.41-0.74) and 0.66 (0.49-0.79) for Wuhan Delta and Omicron variants, respectively, *p<*0.0001; Figure S3]. In line with the findings illustrated above in Figure S2 related to the levels of anti-spike IgG antibodies, the analysis of neutralising antibodies revealed no differences when unvaccinated infected children were stratified by age, gender and presence/absence of symptoms at acute COVID-19, while a lower antibody response were observed in children with comorbidities. Unvaccinated infected children with preexisting diseases were less able to neutralise Wuhan (*p<*0.05) and Delta (*p<*0.01) variants, but neutralised the Omicron variant in a similar fashion compared with children without comorbidities [32 (16-75), 34 (1-63) and 13 (1-32) with comorbidities and 66 (29-123), 65 (30-136) and 14 (1-31) without comorbidities, median (IQR), for Wuhan Delta and Omicron variants, respectively; Figure S4].

### Vaccination of previously infected children increases plasma neutralising activity against SARS-CoV-2 variants

**Figure 2:**
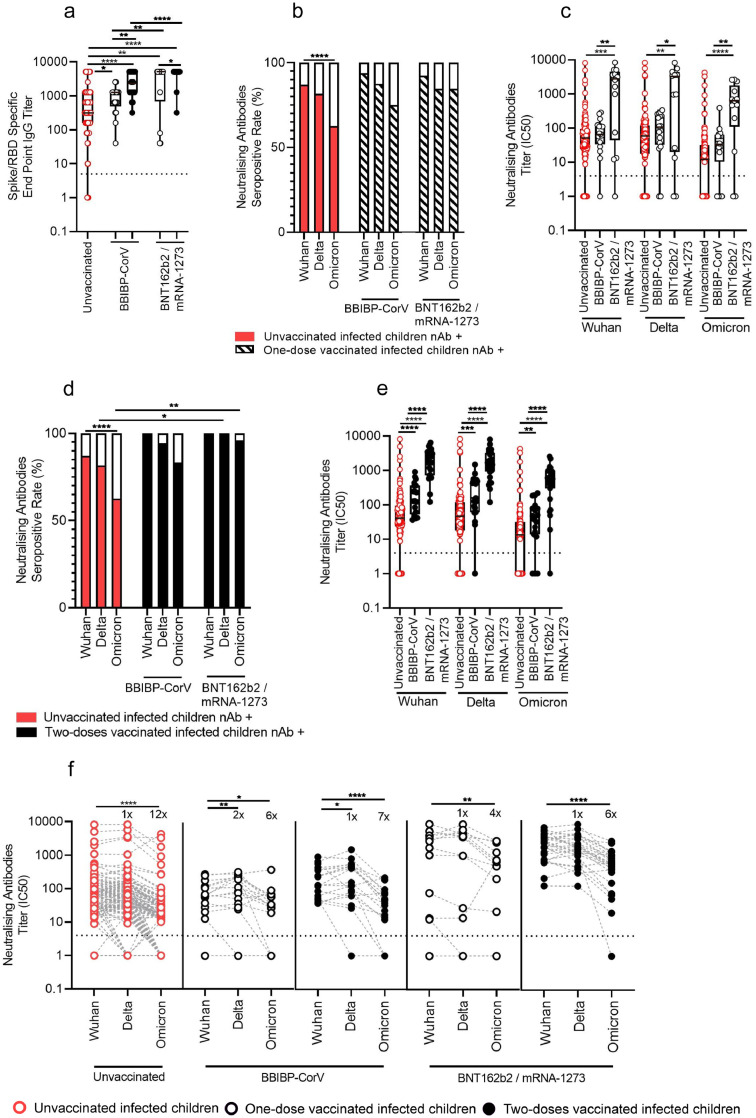
**Antibody response against Wuhan, Delta and Omicron variants in unvaccinated and vaccinated previously SARS-CoV-2 infected children. (a)** Titres of IgG anti-spike antibodies defined by end point dilution in plasma from unvaccinated infected children (*n=*115) and infected children receiving one- (*n=*17) or two-doses of BBIBP-CorV (*n=*19) or one- (*n=*13) or two-doses (*n=*27) of mRNA vaccines. **(b-c)** Neutralising activity against Wuhan, Delta and Omicron variants in plasma from unvaccinated infected and vaccinated previously infected children with one-dose of BBIBP-CorV or mRNA vaccines (BNT162b2 / mRNA-1273). **(b)** Bar graphs show the percentage of positive samples for neutralising activity against Wuhan, Delta and Omicron variants. **(c)** Neutralisation antibody titres against Wuhan, Delta and Omicron variants determined by the reciprocal IC50 in plasma from unvaccinated infected and one-dose vaccinated previously infected children. **(d-e)** Neutralising activity against Wuhan, Delta and Omicron variants in plasma from unvaccinated infected and previously infected children vaccinated with two-doses of BBIBP-CorV or mRNA vaccines. **(d)** Bar graphs show the percentage of positive samples for neutralising activity against Wuhan, Delta and Omicron variants. **(e)** Neutralisation antibody titres against Wuhan, Delta and Omicron variants determined by the reciprocal IC50 in plasma from unvaccinated infected and two-doses vaccinated infected children. **(f)** Comparison of neutralisation antibody titres against the three variants in paired samples. Fold decrease was calculated dividing the Wuhan IC50 by the Delta or Omicron IC50. Dotted line indicates the limit of detection value. Median and min to max of n donors are shown in a, c and e. P values were determined by Pearson's Chi square test and Mann-Whitney U test: * *p<*0.05, ** *p<*0.01, *** *p<*0.001, **** *p<*0.0001. Unvaccinated infected children (red circle), one-dose vaccinated infected children (black circle), two-doses vaccinated infected children (filled black circle).

Plasma levels of anti-spike IgG antibodies and neutralising activity were compared in unvaccinated (*n=*115) and vaccinated infected children (*n=*76) with one or two doses of the COVID-19 vaccines approved for children and adolescents in Argentina: BBIBP-CorV, BNT162b2, and mRNA-1273. A detailed description of the age, vaccination regimen, time since vaccination until sampling, presence of symptoms during acute SARS-CoV-2 infection, comorbidities and antibody titres for each child or adolescent, are shown in Table S1. Considering the small size of the cohort of vaccinated infected children, to analyse the antibody response induced by vaccination we divided children and adolescents into two groups; those receiving the inactivated BBIBP-CorV vaccine and those receiving a mRNA vaccine (BNT162b2, and/or mRNA-1273). Plasma titres of IgG anti-spike antibodies in unvaccinated infected children (*n=*115) showed a median (IQR) of 320 (160-1280). A statistically significant increase in IgG anti-spike titres was observed in infected children vaccinated with one (1280, 480-1280; median, IQR, *n=*17; *p<*0.05) or two-doses of BBIBP-CorV (2560, 1280-5120; median, IQR, *n=*19; *p<*0.0001). Regarding mRNA vaccines, infected children vaccinated with one-dose of these vaccines had higher levels of anti-spike IgG antibodies (5120, 680-5120; median, IQR, *n=*13) than unvaccinated infected children (*p<*0.01). This increase was even higher after the application of a second dose (5120, 5120-5120; median, IQR, *n=*27; *p<*0.0001, Figure 2a). Considering that no vaccines had been approved for children younger than 3 years at the time of the study, no children under this age was included in the vaccinated group. This issue resulted in a significant age discrepancy between vaccinated and non-vaccinated infected children. In view of this, we reanalysed our data after excluding those children under 3 years old in the group of unvaccinated infected children. The median age (IQR) was 8 years (6-13) and 12 years (9-15) for unvaccinated infected and vaccinated infected children, respectively. The results obtained were similar to those observed when both groups were compared without excluding children younger than 3 years (Figure S5)

Analysis of plasma neutralising activity against Wuhan, Delta and Omicron showed no significant differences in the frequency of positive samples from infected children receiving a single dose of either BBIBP-CorV or mRNA vaccines, compared with unvaccinated infected children (Figure 2b). Neutralising titres for Wuhan, Delta and Omicron in unvaccinated infected children (*n=*115) showed a median (IQR) of 52 (24-94), 55 (19-122) and 14 (1-32). Neutralising titres against the three variants increased after vaccination with a single dose of mRNA vaccines [2601 (44-4452), 3398 (20-4389) and 632 (108-1786), median (IQR), *p<*0.001, *p<*0.01 and *p<*0.0001 vs unvaccinated infected children for Wuhan, Delta and Omicron, respectively, *n=*13]. However, application of a single dose of BBIBP-CorV was shown to be unable to increase neutralisation titres [67 (34-119), 114 (33-245) and 35 (10-63), median (IQR) for Wuhan, Delta and Omicron respectively, *n=*17; Figure 2c]. We then studied the neutralising antibody response in infected children and adolescents vaccinated with two doses. Analysis of plasma neutralising activity against Wuhan, Delta and Omicron showed an increase in the frequency of positive samples from children vaccinated with two-doses of either BBIBP-CorV or mRNA vaccines. However, statistically significant differences in the seropositivity were only observed for mRNA vaccines [18% (11-25%, 95% CI), *p<*0.05, and 33% (26-40%, 95% CI), *p<*0.01, for estimated differences between unvaccinated infected vs. vaccinated infected children for Delta and Omicron respectively; Figure 2d). We also found that two-doses of the BBIBP-CorV vaccine increased the titres of neutralising antibodies in vaccinated infected children against the three variants analysed compared with the response of unvaccinated infected children [129 (52-365), 157 (61-511) and 45 (14-79), median (IQR), for Wuhan (*p<*0.001), Delta (*p<*0.001) and Omicron (*p<*0.01) variants, respectively, *n=*19]. As expected, two-doses of mRNA vaccines markedly increased neutralising titres against all the variants, compared with the response of unvaccinated infected children [2255 (724-3456), 1664 (923-3239) and 554 (304-983), median (IQR), for Wuhan (*p<*0.0001), Delta (*p<*0.0001) and Omicron (*p<*0.0001), respectively, *n=* 27; Figure 2e]. Analysis of paired samples further confirmed that neutralising titres against Omicron were markedly lower compared with the titres observed for the Wuhan and Delta variants, in either unvaccinated infected or vaccinated infected children (Figure 2f).

## Discussion

An asymptomatic or mild course is the most frequent outcome of SARS-CoV-2 infection in children. Our observations indicate that upon SARS-CoV-2 infection, children with asymptomatic or mild disease mount a more potent and sustained antibody response when compared to adults with mild disease up to 17 months post infection. Early studies focused on the acute phase of disease or in the early convalescence period suggested that children develop a lower antibody response compared to adults despite similar viral load.^7^^,^^11^^,^^23^^,^^24^ More recently, some studies have shown a persistent and robust immune response to SARS-CoV-2 infection in children.^13^, ^14^, ^15^^,^^25^ All of these studies described that plasma levels of anti-spike IgG antibodies are higher in children compared to adults with mild disease after 2-12 months upon infection, suggesting that children mount a more efficient B cell memory response. However, these studies reported discrepant results when comparing the plasma neutralising activity against SARS-CoV-2 variants, a response that appears to be the best correlate for protection against infection or re-infection.^26^, ^27^, ^28^ Dowell and coworkers showed that children had a higher antibody binding to SARS-CoV-2 VOCs Alpha (B.1.1.7), Beta (B.1.351) and Gamma (P.1) after infection compared to adults but displayed similar neutralising ability to all of these variants^14^. Using a bead-based multiplex immunoassay for 23 human coronavirus antigens including SARS-CoV-2 and its VOCs (Alpha, Beta and Gamma), Renk and coworkers showed that plasma levels of specific antibodies directed to SARS-CoV-2 were increased in children compared to adults and persisted by longer periods. However, no differences were observed in plasma neutralising titres against the Wuhan and Delta variants between children and adults.^15^ Garrido and coworkers, found that 2 and 4 months after infection, children and adolescents showed higher levels of both IgG antibodies directed to different SARS-CoV-2 antigens and neutralising antibodies.^13^ Consistent with this work, by studying young children aged 0-4 years, Karron and coworkers reported that infected children displayed 10-fold increase in the titres of IgG antibodies directed to RBD and 2-fold increase in the titres of neutralising antibodies directed to the Wuhan variant, compared to adults in the same households.^25^ Finally, Bonfante and coworkers, showed that younger children with asymptomatic or mild COVID-19 show higher levels of neutralising antibodies during the first 7 to 8 months after SARS-CoV-2 infection compared with older siblings and adults^29^ while Dowell and coworkers^12^ reported that the IgG antibody response of infected children against the spike protein did not significantly decay when compared at 6 and 12 months after infection. In line with these studies, we found a stronger and more persistent antibody response in unvaccinated infected children with asymptomatic or mild disease compared to unvaccinated infected adults with mild COVID-19.

Virus lineage information was not available for the participants in our cohorts but, in all cases, they were infected between March 2020 and July 2021, before the emergence of the VOC Omicron in our country last December 2021 according to the report of the Proyecto Pais consortium (http://pais.qb.fcen.uba.ar/reports.php).^19^ Most studies related to the escape of Omicron to antibody neutralisation were conducted using sera from adult COVID-19 patients or vaccine recipients.^30^, ^31^, ^32^, ^33^ Importantly, our study reports for the first time that the immune response induced in unvaccinated children after infection by pre-Omicron variants enable them to mount a neutralising antibody response against Omicron. In fact, we found that 60% of unvaccinated infected children had neutralising antibodies against Omicron variant between 7 and 17 months post infection while only 28% of unvaccinated infected adults shared this condition. Moreover, among seropositive samples, the titres of neutralising antibodies were significantly higher in these children compared to adults. As expected, neutralising antibody titres against Omicron were markedly reduced in comparison with the neutralising titres against Wuhan and Delta variants in both, unvaccinated infected children and adults.

Our study also focused on the impact of vaccination on the antibody response in previously infected children. To our best knowledge, no previous study has been published regarding this issue. However, studies performed in adults have shown that hybrid immunity (i.e., immunity conferred by previous infection plus vaccination) induces a more sustained immune response and provides better protection against infection compared with vaccination or infection alone.^34^ Vaccination with BBIBP-CorV was approved in Argentina for children of 3-11 years in October 2021, while BNT162b2 and mRNA-1273 were approved for children and adolescents of 12-17 years, in August 2021. We found that both types of vaccines enhanced the antibody response in previously infected children. However, mRNA vaccines induced higher titres of either anti-spike IgG antibodies and neutralising antibodies against the Wuhan, Delta and Omicron variants, compared with BBIBP-CorV. The reduced response elicited by BBIBP-CorV could be explained not only by a lower immunogenicity of this vaccine compared with mRNA vaccines, but also by the younger age of the children vaccinated with BBIBP-CorV in our country. As expected, analysis of paired samples of vaccinated infected children showed that neutralising titres against Omicron were markedly lower compared with the titres against Wuhan and Delta variants. By studying a cohort of 15 unvaccinated convalescent children, Chen and coworkers^35^ reported that only 27% of unvaccinated convalescents showed neutralising antibodies against the Omicron variant. This observation contrast with our findings showing that 63% of unvaccinated infected children had neutralising antibodies against Omicron. This could be explained, at least in part, by differences in the variants responsible for infection as well as for the different time elapsed between infection and sample collection in each of the studies.

This study has a number of limitations. Firstly, the stratified analysis by age, gender, the presence of comorbidities and the time elapsed between infection and sampling need to be carefully interpreted due to the relatively small sample sizes included in our study. It is also important to emphasise that our cohort did not include children and adults with moderate or severe COVID-19. Additionally, we were not able to obtain paired samples of unvaccinated children during early convalescence or immediately before vaccination. Although children with SARS-CoV-2 re-infection were excluded from our study, we cannot rule out that some children may have suffered an asymptomatic re-infection before sampling. The fact that the BBIBP-CorV vaccine and mRNA vaccines have been approved in our country for different age groups makes it difficult to compare them in terms of immunogenicity. Moreover, since no vaccine had been approved for children younger than 3 years at the time of this study, the comparison between the antibody response of unvaccinated and vaccinated children presents a significant age bias. Finally, the small volume of blood collected from each patient precluded detailed studies aimed at analysing the antigen-specific B and T memory cells.

Our observations suggest that although children and youth are less severely affected by SARS-CoV-2 infection in most cases, they develop a strong and more sustained antibody immune response in comparison with adults. Importantly, the ability to mount a more vigorous antibody response is clearly expressed in the development of a higher antibody response against the Omicron variant following infections by pre-Omicron variants. As expected, vaccination of previously infected children with either BBIBP-CorV or mRNA vaccines further increased the antibody response, although mRNA vaccines were shown to be more immunogenic. Our findings could contribute not only to better understanding the immune response of children against SARS-CoV-2 infection, but also to better define guidelines for vaccination in children and adolescents. Further studies are needed to define the anti-SARS-CoV-2 vaccination strategy in previously infected children. These studies should include not only the assessment of the immune response elicited by vaccines but also their effectiveness in preventing both SARS-CoV-2 infection and disease severity.

## Contributors

Conception and design: V.S., S.R., J.G., and L.A.; Enrollment of donors, collection of blood sample and clinical data: S.R., M.B., L.H., M.U., M.M.P., C.C., C.E., L.S.A., M.L., L.D.M., C.D., A.A.F., S.H.A., Y.R., L.M., L.S., M.G., Y.L., N.D.C., M.M.B., N.L., F.F., and L.A.; Performance of lab experiments: V.S., C.R., A.V., I.M., F.D.D., B.M., I.S., A.C., and L.A.; Analysis and interpretation: V.S., S.R., J.M.G.P, N.L., J.G. and L.A.; Drafting the manuscript for significant intellectual content: V.S., J.G., and L.A. All authors contributed, verified the underlying data and approved the submitted version of the manuscript.

## Data sharing statement

Any data not published within the article will be shared in anonymised format by request from any qualified investigator. If desired, please contact the corresponding author of this article.

## Declaration of interests

The authors have nothing to disclose.

## Appendix Supplementary materials

Image, application 1
