# Supplementary material for: Antibody response against SARS-CoV-2 variants of concern in children infected with pre-Omicron variants: An observational cohort study
Source: eBioMedicine. 2022 Aug 18;83:104230. doi: 10.1016/j.ebiom.2022.104230 (PMC9387350; doi:10.1016/j.ebiom.2022.104230)
Supplement: Supplementary file 1 [file mmc1.docx]

**Table S1. Characteristics of vaccinated previously infected children**

| Patient | Age (y) | Vaccination regimen | Doses administered^a^ | Days post-vaccination until sampling^b^ | With symptoms^c^ | With comorbidities | SARS-CoV-2 IgG titer | Neutralising titer (IC50) | | |
| --- | --- | --- | --- | --- | --- | --- | --- | --- | --- | --- |
|  |  |  |  |  |  |  |  | Wuhan | Delta | Omicron |
| 1 | 10 | BBIBP-CorV | 1 | 16 | No | Yes | 640 | 115 | 229 | 67 |
| 2 | 5 | BBIBP-CorV | 1 | 16 | Yes | Yes | 1280 | 123 | 260 | 58 |
| 3 | 10 | BBIBP-CorV | 1 | 23 | Yes | No | 320 | 62 | 269 | 1 |
| 4 | 11 | BBIBP-CorV | 1 | 24 | No | No | 160 | 1 | 1 | 1 |
| 5 | 3 | BBIBP-CorV | 1 | 18 | Yes | No | 640 | 58 | 85 | 35 |
| 6 | 9 | BBIBP-CorV | 1 | 30 | No | Yes | 640 | 110 | 117 | 33 |
| 7 | 6 | BBIBP-CorV | 1 | 28 | Yes | Yes | 1280 | 187 | 222 | 59 |
| 8 | 10 | BBIBP-CorV | 1 | 15 | Yes | No | 1280 | 61 | 78 | 29 |
| 9 | 11 | BBIBP-CorV | 1 | 15 | No | No | 640 | 67 | 201 | 27 |
| 10 | 9 | BBIBP-CorV | 1 | 28 | Yes | No | 2560 | 289 | 294 | 393 |
| 11 | 7 | BBIBP-CorV | 1 | 19 | Yes | Yes | 1280 | 71 | 52 | 71 |
| 12 | 11 | BBIBP-CorV | 1 | 28 | Yes | Yes | 40 | 13 | 1 | 1 |
| 13 | 11 | BBIBP-CorV | 1 | 21 | Yes | No | 1280 | 29 | 37 | 20 |
| 14 | 11 | BBIBP-CorV | 1 | 15 | Yes | No | 320 | 39 | 25 | 1 |
| 15 | 3 | BBIBP-CorV | 1 | 142 | Yes | Yes | 1280 | 21 | 29 | 94 |
| 16 | 5 | BBIBP-CorV | 1 | 50 | No | Yes | 1280 | 263 | 337 | 36 |
| 17 | 5 | BBIBP-CorV | 1 | 127 | Yes | No | 2560 | 112 | 114 | 58 |
| 18 | 14 | BNT162b2 | 1 | 12 | Yes | No | 5120 | 3647 | 8192 | 2641 |
| 19 | 15 | BNT162b2 | 1 | 16 | Yes | No | 5120 | 5142 | 3398 | 482 |
| 20 | 16 | BNT162b2 | 1 | 18 | Yes | No | 5120 | 3812 | 4491 | 697 |
| 21 | 17 | BNT162b2 | 1 | 30 | Yes | No | 5120 | 995 | 956 | 465 |
| 22 | 14 | BNT162b2 | 1 | 47 | Yes | No | 5120 | 3153 | 3599 | 632 |
| 23 | 14 | BNT162b2 | 1 | 30 | Yes | Yes | 40 | 1 | 1 | 1 |
| 24 | 13 | BNT162b2 | 1 | 19 | Yes | No | 80 | 13 | 14 | 708 |
| 25 | 14 | BNT162b2 | 1 | 25 | Yes | No | 1280 | 74 | 27 | 21 |
| 26 | 15 | BNT162b2 | 1 | 26 | Yes | No | 40 | 12 | 1 | 1 |
| 27 | 12 | BNT162b2 | 1 | 23 | Yes | No | 5120 | 2601 | 5585 | 1202 |
| 28 | 15 | mRNA-1273 | 1 | 14 | Yes | No | 5120 | 8353 | 4286 | 2369 |
| 29 | 13 | mRNA-1273 | 1 | 22 | No | Yes | 5120 | 5092 | 3604 | 2617 |
| 30 | 14 | mRNA-1273 | 1 | 103 | Yes | Yes | 5120 | 1639 | 980 | 196 |
| 31 | 10 | BBIBP-CorV | 2 | 77 | Yes | No | 1280 | 85 | 61 | 77 |
| 32 | 3 | BBIBP-CorV | 2 | 44 | Yes | Yes | 1280 | 52 | 1 | 1 |
| 33 | 4 | BBIBP-CorV | 2 | 95 | Yes | Yes | 5120 | 245 | 485 | 189 |
| 34 | 9 | BBIBP-CorV | 2 | 33 | Yes | No | 1280 | 359 | 557 | 32 |
| 35 | 11 | BBIBP-CorV | 2 | 77 | Yes | Yes | 5120 | 653 | 1496 | 185 |
| 36 | 11 | BBIBP-CorV | 2 | 47 | Yes | No | 5120 | 128 | 157 | 22 |
| 37 | 6 | BBIBP-CorV | 2 | 66 | No | No | 5120 | 47 | 32 | 17 |
| 38 | 10 | BBIBP-CorV | 2 | 48 | Yes | No | 2560 | 62 | 128 | 1 |
| 39 | 6 | BBIBP-CorV | 2 | 62 | No | No | 1280 | 566 | 91 | 38 |
| 40 | 11 | BBIBP-CorV | 2 | 41 | Yes | No | 2560 | 229 | 511 | 45 |
| 41 | 11 | BBIBP-CorV | 2 | 30 | Yes | No | 2560 | 435 | 535 | 96 |
| 42 | 5 | BBIBP-CorV | 2 | 19 | Yes | Yes | 5120 | 129 | 123 | 218 |
| 43 | 5 | BBIBP-CorV | 2 | 16 | Yes | No | 1280 | 40 | 26 | 1 |
| 44 | 9 | BBIBP-CorV | 2 | 39 | Yes | Yes | 320 | 42 | 60 | 14 |
| 45 | 11 | BBIBP-CorV | 2 | 35 | No | No | 1280 | 122 | 410 | 69 |
| 46 | 3 | BBIBP-CorV | 2 | 43 | Yes | No | 640 | 37 | 81 | 12 |
| 47 | 3 | BBIBP-CorV | 2 | 29 | Yes | Yes | 5120 | 244 | 424 | 50 |
| 48 | 4 | BBIBP-CorV | 2 | 30 | Yes | No | 5120 | 906 | 803 | 79 |
| 49 | 9 | BBIBP-CorV | 2 | 55 | Yes | Yes | 640 | 365 | 465 | 48 |
| 50 | 12 | BNT162b2 | 2 | 38 | No | No | 5120 | 3070 | 3996 | 920 |
| 51 | 17 | BNT162b2 | 2 | 16 | Yes | Yes | 5120 | 724 | 1014 | 1 |
| 52 | 14 | BNT162b2 | 2 | 78 | Yes | No | 5120 | 4203 | 4096 | 1210 |
| 53 | 16 | BNT162b2 | 2 | 49 | Yes | No | 5120 | 3353 | 2912 | 2210 |
| 54 | 16 | BNT162b2 | 2 | 74 | Yes | Yes | 5120 | 2255 | 923 | 64 |
| 55 | 16 | BNT162b2 | 2 | 46 | Yes | No | 5120 | 1042 | 1400 | 304 |
| 56 | 12 | BNT162b2 | 2 | 46 | Yes | No | 5120 | 1191 | 1877 | 554 |
| 57 | 13 | BNT162b2 | 2 | 115 | Yes | No | 5120 | 1708 | 1891 | 381 |
| 58 | 15 | BNT162b2 | 2 | 113 | Yes | No | 5120 | 582 | 436 | 71 |
| 59 | 13 | BNT162b2 | 2 | 116 | Yes | Yes | 5120 | 3456 | 2112 | 983 |
| 60 | 15 | BNT162b2 | 2 | 61 | Yes | No | 5120 | 5444 | 3463 | 687 |
| 61 | 12 | BNT162b2 | 2 | 54 | Yes | No | 5120 | 202 | 377 | 49 |
| 62 | 15 | mRNA-1273 | 2 | 41 | Yes | Yes | 5120 | 718 | 288 | 362 |
| 63 | 16 | mRNA-1273 | 2 | 16 | Yes | No | 5120 | 3716 | 1799 | 532 |
| 64 | 16 | mRNA-1273 | 2 | 37 | No | Yes | 5120 | 5083 | 1479 | 569 |
| 65 | 14 | mRNA-1273 | 2 | 26 | Yes | Yes | 5120 | 4021 | 5772 | 2559 |
| 66 | 12 | mRNA-1273 | 2 | 26 | Yes | Yes | 5120 | 3035 | 1951 | 794 |
| 67 | 14 | mRNA-1273 | 2 | 22 | Yes | Yes | 5120 | 6432 | 7994 | 991 |
| 68 | 16 | mRNA-1273 | 2 | 69 | Yes | Yes | 5120 | 2590 | 4119 | 1270 |
| 69 | 16 | mRNA-1273 | 2 | 78 | Yes | Yes | 5120 | 1699 | 3239 | 610 |
| 70 | 17 | mRNA-1273 | 2 | 73 | Yes | No | 5120 | 626 | 1664 | 483 |
| 71 | 16 | mRNA-1273 | 2 | 42 | Yes | Yes | 5120 | 615 | 452 | 603 |
| 72 | 17 | mRNA-1273 | 2 | 40 | Yes | No | 5120 | 1685 | 636 | 147 |
| 73 | 15 | mRNA-1273 | 2 | 123 | Yes | Yes | 5120 | 2355 | 1137 | 991 |
| 74 | 12 | mRNA-1273 | 2 | 58 | No | No | 320 | 1623 | 1012 | 433 |
| 75 | 15 | mRNA-1273 | 2 | 23 | Yes | No | 5120 | 2584 | 1219 | 487 |
| 76^d^ | 16 | mRNA-1273 / BNT162b2 | 2 | 15 | Yes | No | 1280 | 121 | 119 | 19 |

^a^ At time of sampling; ^b^ time since first or second dose was administered until sampling; ^c^ in the course of acute infection; ^d^ patient #76 received mRNA-1273 as first dose and BNT162b as second dose.

**Figure S1**

**
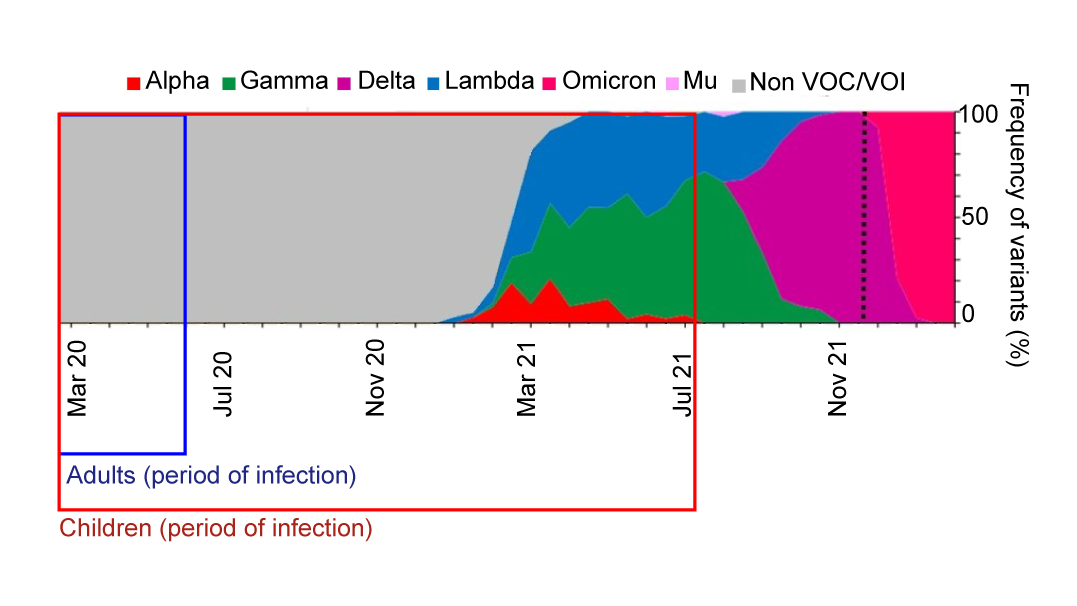
**

**Legend to Figure S1. SARS-CoV-2 major lineages distribution in Argentina over the period of March 2020 to February 2022 and the period of infection of the children and adults cohorts included in the study.** An overlay between the frequency of SARS-CoV-2 variants with or without mutations of interest circulating in Argentina since March 2020 until February 2022 and the period of infection of the unvaccinated and vaccinated children and unvaccinated adults cohorts is shown in the chart. Dotted line shows that Omicron variant has been detected for first time in our country in December 2021. Surveillance of SARS-CoV-2 variants data have been published by Proyecto PAIS consortium (http://pais.qb.fcen.uba.ar/).

**Figure S2**


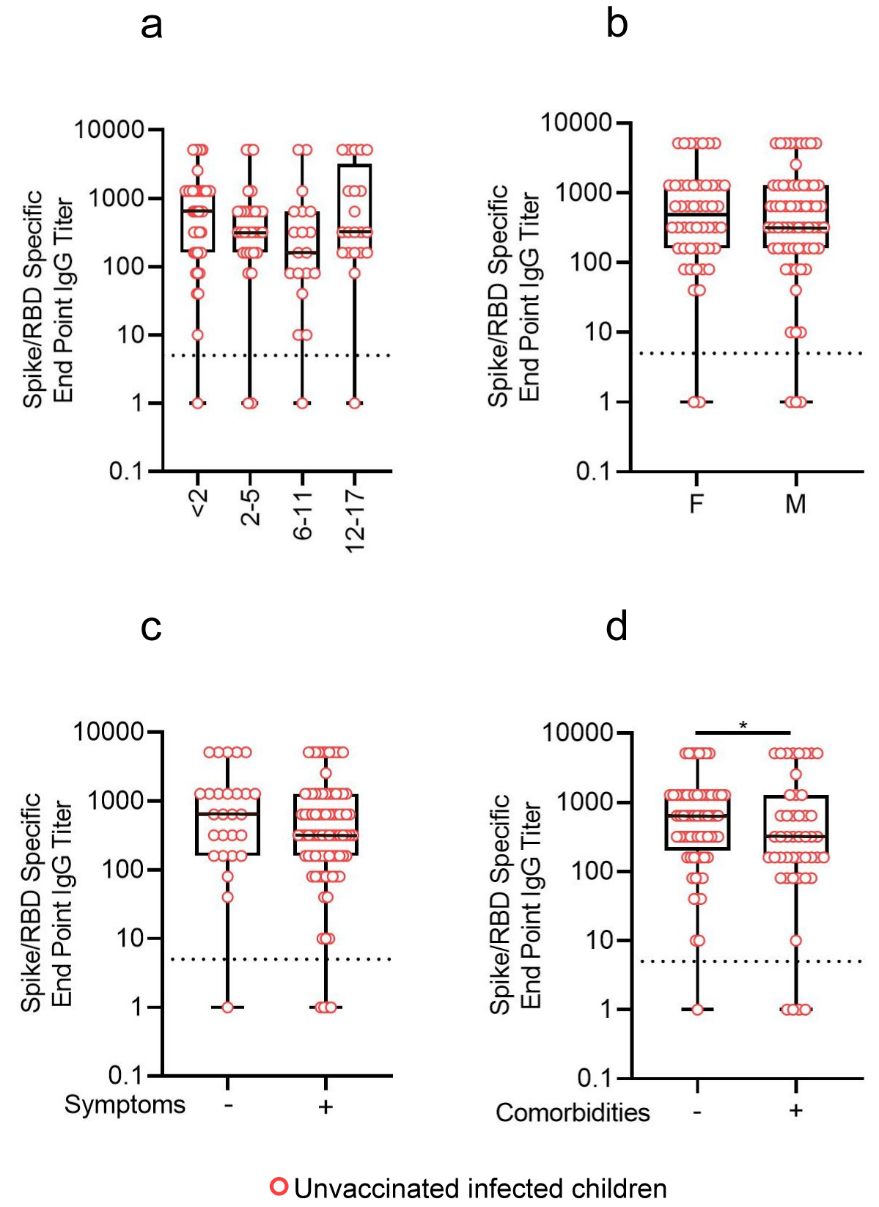


**Legend to Figure S2. Antibody response against SARS-CoV-2 in unvaccinated infected children stratified according to age, gender, symptoms at acute COVID-19 and comorbidities. (a-d)** Plasma titers of anti-spike IgG antibodies in unvaccinated infected children are shown. **(a)** <2 years, n=44; 2 to 5 years, n=30; 6 to 11 years, n=26; and 12-17 years, n=15. **(b)** Girls, n=50 and boys, n=65. **(c)** With symptoms, n=89 and without symptoms, n=26. **(d)** With comorbidities, n=46 and without comorbidities, n=69. Dotted line indicates the limit of detection value. Median and min to max of n donors are shown. P values were determined by Kruskal-Wallis test and Mann-Whitney U test: * p<0.05.

**Figure S3**


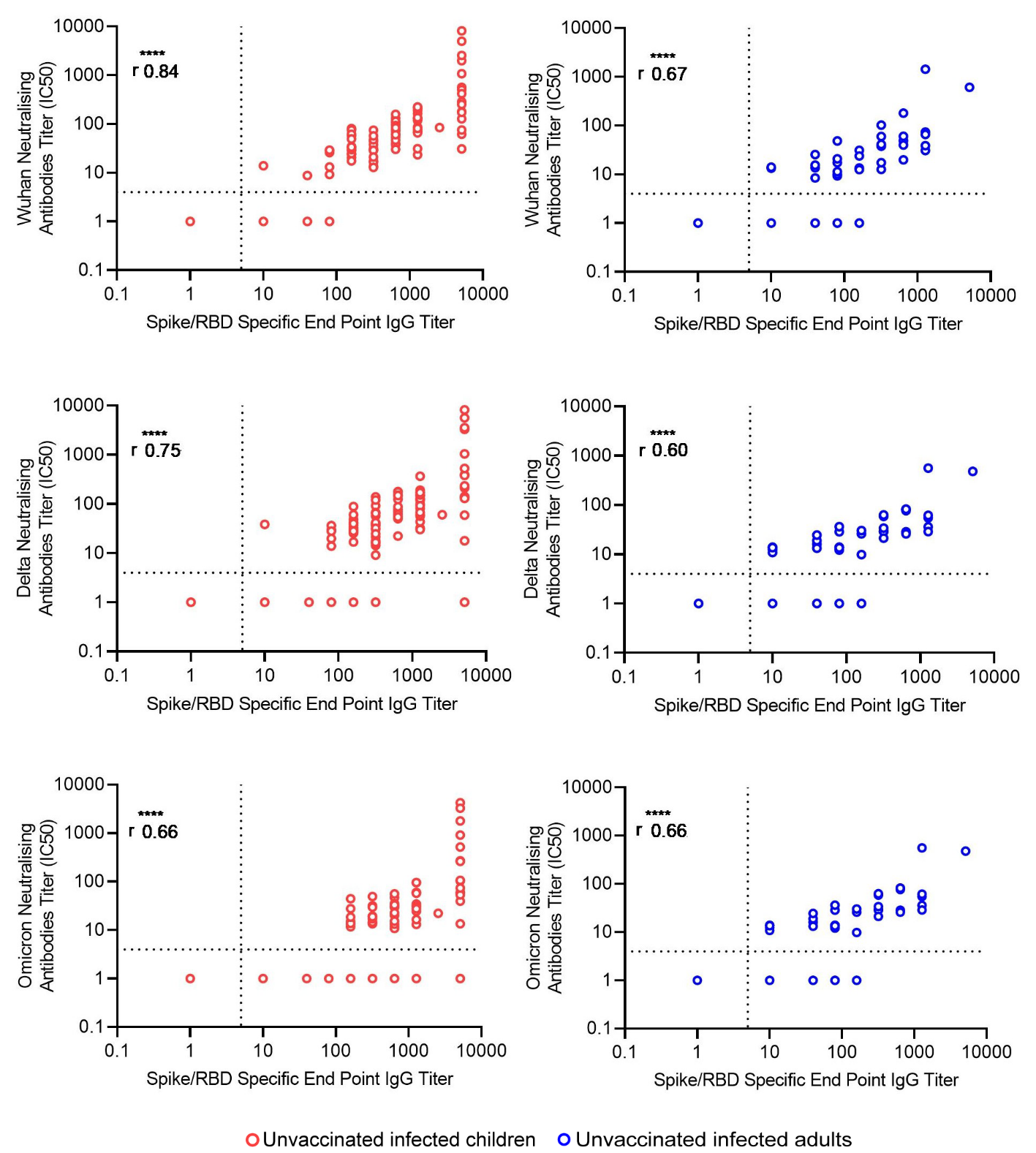


**Legend to Figure S3. Correlation between the titers of anti-spike IgG antibodies and neutralising antibody titers against Wuhan, Delta and Omicron variants in plasma from unvaccinated infected children and unvaccinated infected adults.** Unvaccinated infected children (n=115) and unvaccinated infected adults (n=62). Dotted lines indicate the limit of detection values. P values were determined by Spearman correlation test: **** p<0.0001. Unvaccinated infected children (red circle), unvaccinated infected adults (blue circle).

**Figure S4**

**
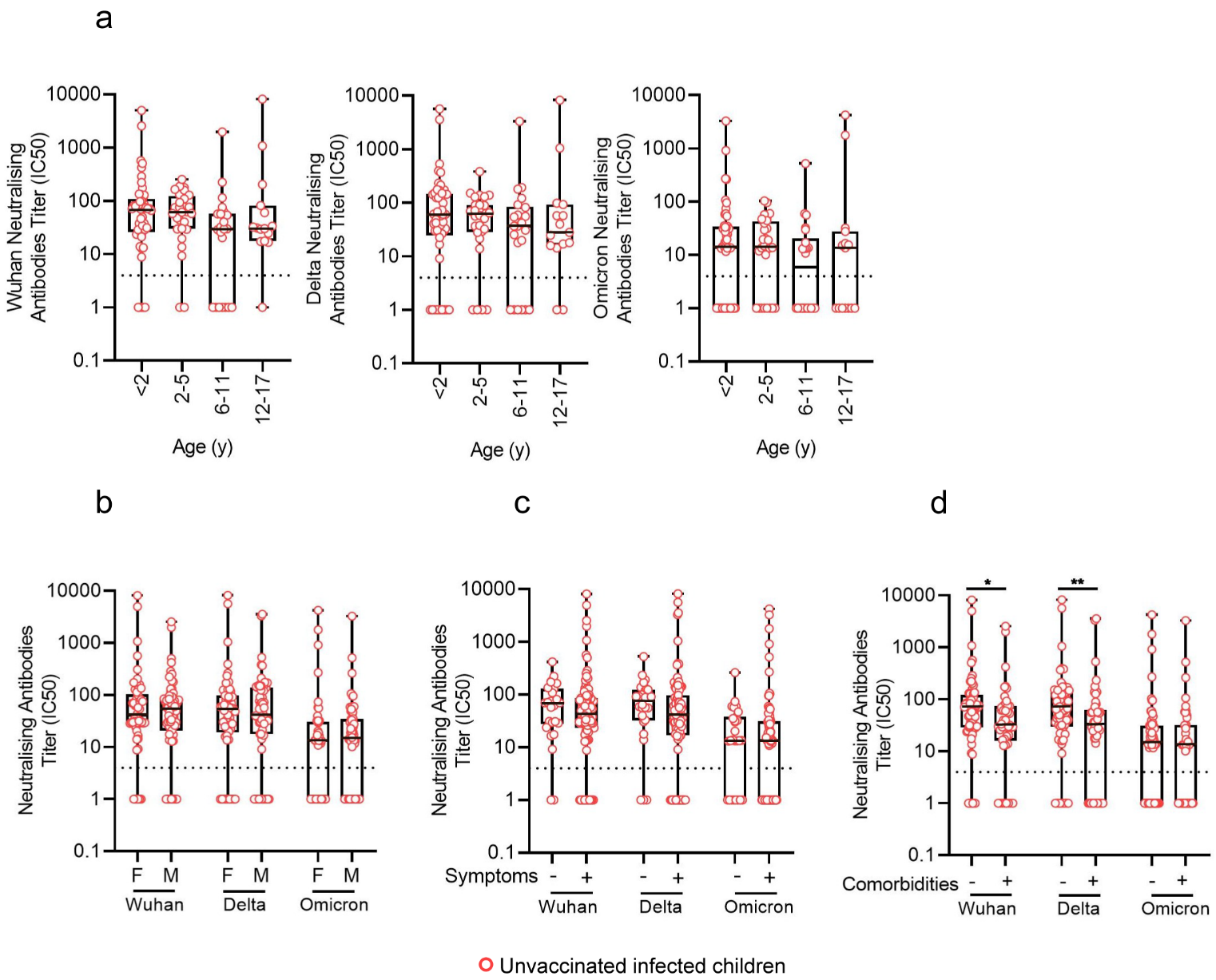
**

**Legend to Figure S4. Neutralising antibody titers against Wuhan, Delta and Omicron variants in unvaccinated infected children stratified according to age, gender, symptoms at acute COVID-19 and comorbidities. (a-d)** Neutralising antibody titers determined by the reciprocal IC50 in unvaccinated infected children are shown. **(a)** <2 years, n=44; 2 to 5 years, n=30; 6 to 11 years, n=26; and 12-17 years, n=15. **(b)** Girls, n=50 and boys, n=65. **(c)** With symptoms, n=89 and without symptoms, n=26. **(d)** With comorbidities, n=46 and without comorbidities, n=69. Dotted line indicates the limit of detection value. Median and min to max of n donors are shown. P values were determined by Kruskal-Wallis test and Mann-Whitney U test: * p<0.05, ** p<0.01.

**Figure S5**

**
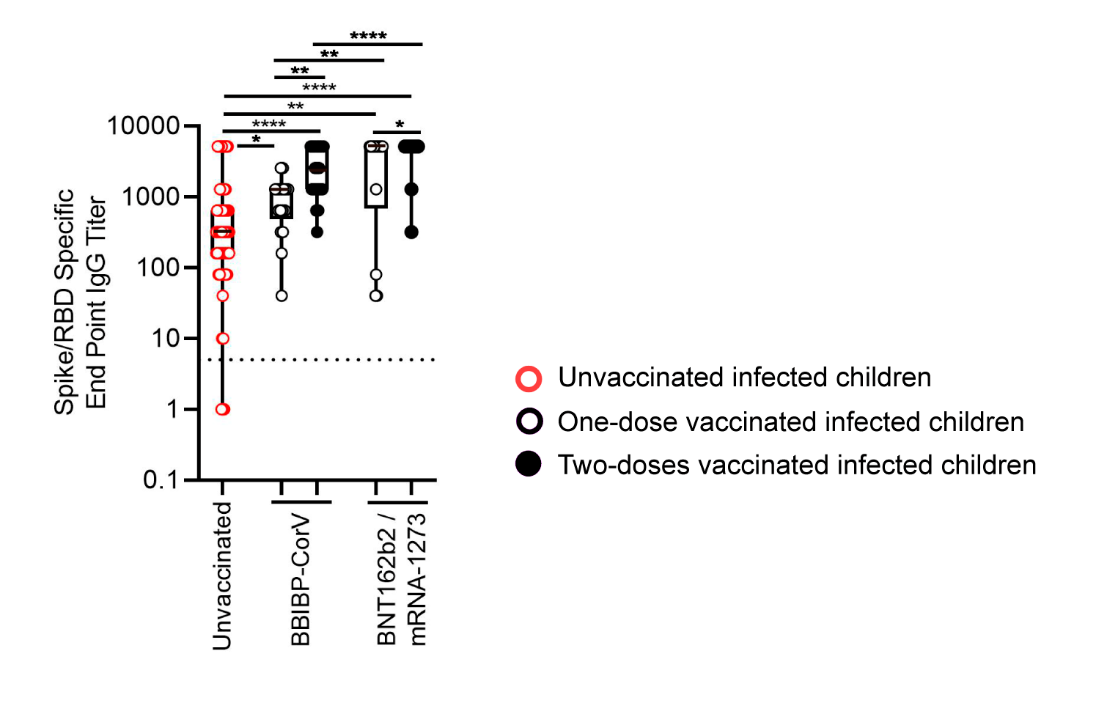
**

**Legend to Figure S5. Antibody response in unvaccinated and vaccinated previously infected children.** Titers of IgG anti-spike antibodies defined by end point dilution in plasma from unvaccinated infected children aged between 3 and 17 years (n=55) and infected children receiving one- (n=17) or two-doses of BBIBP-CorV (n=19) and one- (n=13) or two-doses (n=27) of mRNA vaccines. Dotted line indicates the limit of detection value. Median and min to max of n donors are shown. P values were determined by Kruskal-Wallis test and Mann-Whitney U test: * p<0.05, ** p<0.01, ****p<0.0001. Unvaccinated infected children (red circle), one-dose vaccinated infected children (black circle), two-dose vaccinated infected children (filled black circle).
